# On Global Quantum Communication Networking

**DOI:** 10.3390/e22080831

**Published:** 2020-07-29

**Authors:** Ivan B. Djordjevic

**Affiliations:** Department of Electrical and Computer Engineering, University of Arizona, Tucson, AZ 85721, USA; ivan@email.arizona.edu; Tel.: +1-520-626-5119

**Keywords:** quantum key distribution (QKD), discrete variable (DV)-QKD, continuous variable (CV)-QKD, postquantum cryptography (PQC), quantum communications networks (QCNs)

## Abstract

Research in quantum communications networks (QCNs), where multiple users desire to generate or transmit common quantum-secured information, is still in its beginning stage. To solve for the problems of both discrete variable- and continuous variable-quantum key distribution (QKD) schemes in a simultaneous manner as well as to enable the next generation of quantum communication networking, in this Special Issue paper we describe a scenario where disconnected terrestrial QCNs are coupled through low Earth orbit (LEO) satellite quantum network forming heterogeneous satellite–terrestrial QCN. The proposed heterogeneous QCN is based on the cluster state approach and can be used for numerous applications, including: (i) to teleport arbitrary quantum states between any two nodes in the QCN; (ii) to enable the next generation of cyber security systems; (iii) to enable distributed quantum computing; and (iv) to enable the next generation of quantum sensing networks. The proposed QCNs will be robust against various channel impairments over heterogeneous links. Moreover, the proposed QCNs will provide an unprecedented security level for 5G+/6G wireless networks, Internet of Things (IoT), optical networks, and autonomous vehicles, to mention a few.

## 1. Introduction

Quantum communication (QuCom) employs quantum information theory concepts, in particular the no-cloning theorem and the theorem of indistinguishability of arbitrary quantum states, to implement the distribution of keys with verifiable security, commonly referred to as quantum key distribution (QKD), where security is guaranteed by the fundamental laws of physics as opposed to unproven mathematical assumptions employed in computational security-based cryptography [1,2,3]. Despite the appealing features of QuComs, there are some fundamental and technical challenges that need to be addressed prior to its widespread application. For instance, both the rate and distance of QuCom are fundamentally limited by channel loss, which is specified by the rate-loss tradeoff. To overcome the rate-distance limit of discrete variable (DV)-QKD protocols, two predominant approaches have been pursued recently: (i) the development of quantum relays and (ii) the employment of trusted relays. Quantum relays require the use of long-duration quantum memories and high-fidelity entanglement distillation [4], which are not yet widely available. On the other hand, the trusted-relay methodology assumes that the relay between two users can be trusted [5]; unfortunately, this assumption is difficult to verify in practice. The measurement device independent (MDI)-QKD approach [6] was able to close the detection loopholes; however, its secret-key rate (SKR) is still bounded by *O*(*T*)-dependence (with *T* standing for transmissivity). Recently, twin-field (TF) QKD has been proposed to overcome the rate-distance limit [7], whose SKR scales with the square-root of transmittance, which represents a promising approach to extend the transmission distance. Another key limitation of DV-QKD is the deadtime of single-photon detectors (SPDs), which limits the baud rate and consequently the SKRs. To solve for this problem, a continuous variable (CV)-QKD can be used instead [1,8,9,10], which employs homodyne/heterodyne detection instead and thus does not exhibit the SPDS’ deadtime limitation problem. In particular, the discrete modulation (DM)-based CV-QKD protocols offer much better reconciliation efficiency compared to that of Gaussian modulation (GM)-based CV-QKD protocols. Unfortunately, the security proofs of DM-based CV-QKD schemes for collective and coherent attacks are still incomplete. To overcome key challenges for DV-QKD, such as low SKR values and limited distance, as well as for DM-based CV-QKD, such as incompleteness of security proofs, the following approaches have been proposed in our recent papers: (1) discretized GM (DGM)-CV-QKD [11], (2) optimized CV-QKD [12], and (3) hybrid DV-CV QKD [13]. An alternative approach to QKD is post-quantum cryptography (PQC) [14]. PQC is typically referred to by various cryptographic algorithms that are thought to be secure against any quantum computer-based attack. Unfortunately, PQC is also based on unproven assumptions and some of the PQC algorithms will be broken in the future by developing more sophisticated quantum algorithms.

Modern classical communication networks consist of multiple nodes connected by various types of channels, including free-space optical (FSO) links, optical fibers, ground–satellite links, wireless RF, and coaxial cables. Such a heterogeneous architecture would be equally important for QCNs, as quantum nodes may access a QCN via different kinds of channels. Indeed, quantum communications have been individually validated in free-space, optical fibers, and between a satellite and a ground station, but a combined heterogeneous QCN employing multiple types of channels remains elusive. Unlike in the point-to-point communication case, the fundamental quantum communication rate limits are not well known. Several QKD testbeds have been reported so far, including the DARPA QKD network [15], Tokyo QKD network [16], and secure communication based on quantum cryptography (SECOQC) network [17]. The QKD can also be used to establish QKD-based campus-to-campus virtual private networks employing the IPsec protocol [18] as well as to establish the network setup for using transport-layer security (TLS) based on QKD [19]. However, all of these networks employ the dark fiber infrastructure. Quantum communication over satellite links has already been demonstrated; see for example [20,21]. 

In this Special Issue paper, we propose to implement the multipartite QCN by employing the cluster state-based concept [22]. The proposed quantum network can be used to: (i) perform distributed quantum computing, (ii) teleport quantum states between any two nodes in the network, and (iii) enable the next generation of cyber security systems. The cluster states can be described by using the stabilizer formalism and as such they can easily be certified by simple syndrome measurements. In this formalism, the cluster states can be interpreted as codewords of a corresponding quantum error correction code, while corresponding errors can be corrected for by simple syndrome decoding, among others. By performing simple Y and Z measurements on properly selected nodes we can straightforwardly establish the Einstein–Podolsky–Rosen (EPR) pair between any two nodes in the network. Moreover, multiple EPR pairs can be established simultaneously. We further propose a cluster state-based quantum network of satellites that enables global coverage. The quantum satellite network would be composed of quantum subnetworks comprised of low Earth orbit (LEO) satellites. Some of these LEO satellite-based quantum subnetworks can be connected to a subnetwork of medium Earth orbit (MEO)/ geostationary orbit (GEO) satellites. The LEO satellites should be used to interconnect terrestrial cluster state-based quantum networks. This quantum global network can also be used to distribute the entangled states for quantum sensing applications and to enable distributed quantum computing on a global scale. SDN concepts should be used to reconfigure the proposed QCN.

The paper is organized as follows. In Section 2, we describe the proposed cluster states-based QCN concept. In Section 3, we describe potential approaches to extend the transmission distance between QCN nodes. In Section 4, we describe the QCN that is currently under development at the University of Arizona. Finally, in Section 5, we provide some relevant concluding remarks.

## 2. Proposed Cluster States-Based Quantum Communications Networks

To enable the next generation of quantum communication networking, we envision a scenario in which disconnected terrestrial cluster states-based QCNs are coupled through the LEO satellite (cluster state) quantum network, thus providing global coverage. The proposed quantum network will be highly robust against turbulence encountered by FSO links, as the envisioned quantum satellite network will communicate to ground nodes only through the LEO satellite-to-ground links, exhibiting a vertical downlink profile through vacuum followed by a turbulence layer with strength that is altitude-dependent. 

**Figure 1:**
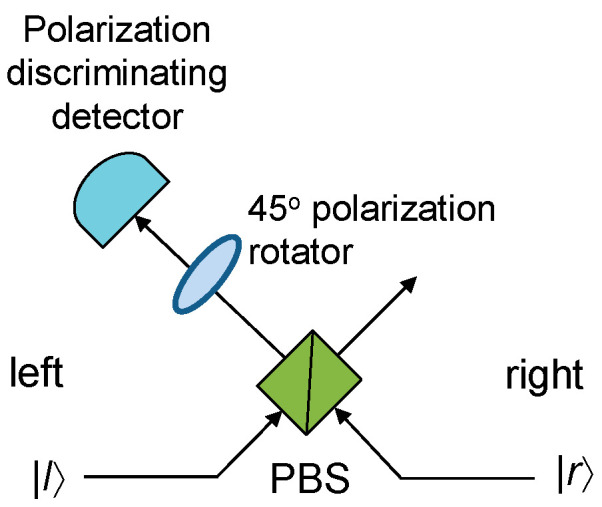
Illustrating the type I fusion process. PBS: polarization beam splitter.

**Figure 2:**
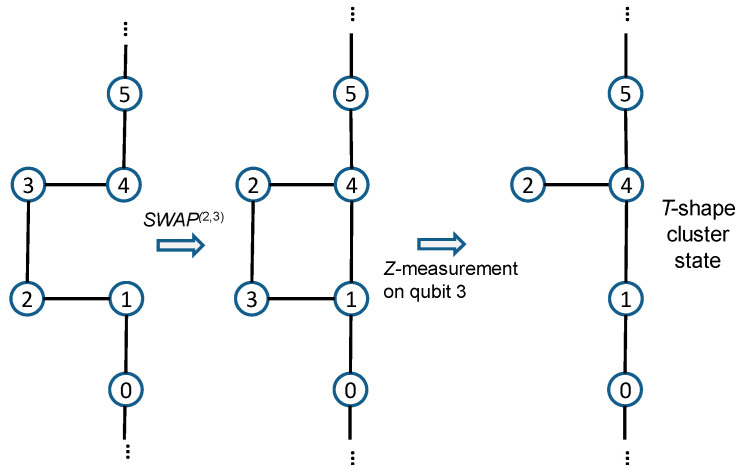
Gilbert’s approach to create the *T*-shape cluster state.

The cluster states belong to the class of the graph states, which also include Bell states, Greenberger–Horne–Zeilinger (GHZ) states, W-states, and various entangled states used in quantum error correction [22]. When the cluster *C* is defined as a connected subset on a *d*-dimensional lattice, it obeys the set of eigenvalue equations $S_{a}{|\phi \rangle }_{C}={|\phi \rangle }_{C},\;S_{a}=X_{a}\underset{b\in N\left(a\right)}{⊗}Z_{b}$, where *S_a_* are *stabilizer operators* with *N*(*a*) denoting the neighborhood of *a* ∈ *C*. To create a 2-D cluster state, the approach proposed by Gilbert et al. [23] is applicable; it employs linear states, generated by spontaneous parametric down conversion (SPDC), local unitaries, and type I fusion to create the desired 2-D cluster state. The type I fusion is illustrated in Figure 1, based on [23]. The vertical photon is reflected by the polarization beam splitter (PBS), while the horizontal photon is transmitted through the PBS. Given the probabilistic nature of the PBS, with the photons present at both the left and right input ports, there are four possible outcomes, each occurring with probability 0.25. Two outcomes correspond to the desired fusion operators, and the success probability of the fusion is 0.5. When a single photon is detected by the detector, a successful fusion is declared. The procedure to create the T-shape cluster state is described in Figure 2. To create the box-cluster state, we start with a four-qubit linear cluster state, re-label the qubits 2 and 3, and apply the Hadamard gates to qubits 2 and 3, which effectively establish the bond between qubits 1 and 4. Namely, relabeling the qubits is equivalent to the SWAP gate action. To create the box-on-chain cluster state, we start with a longer linear chain of qubits and apply the same approach as in a box-state creation. Two T-shape cluster states can be fused together to get the *H*-shape cluster state, etc.

**Figure 3:**
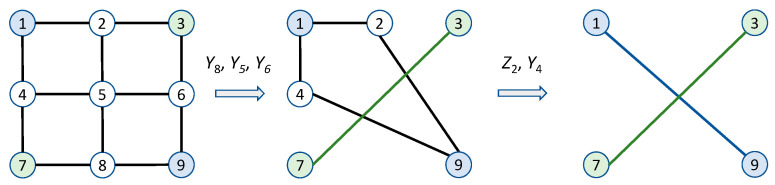
Establishing EPR pairs between nodes 1 and 9 as well as between nodes 3 and 7.

Once the 2-D cluster state of nodes is created, we can use properly selected *Y* and *Z* measurements to create the EPR pair between any two arbitrary nodes in the quantum network. As a reminder, the role of the *Z* measurement is to remove the particular node (qubit) from the cluster, whereas the role of *Y* measurement is to remove a given node and link neighboring nodes. As an illustration, the 2-D cluster state with nine nodes is shown in Figure 3. Let us assume that we are interested in establishing EPR pairs between nodes 3 and 7 as well as nodes 1 and 9. We first perform *Y* measurements in the following order: *Y*_8_, *Y*_5_, and *Y*_6_ to get the intermediate stage. We then perform *Z*-measurement on node 2 and Y measurement on node 4 to get the two desired EPR pairs. Given that the 2-D cluster state is universal, it is possible to use the same network architecture for both QCN and distributed quantum computing. We also imagine the scenario in which each node is equipped with multiple qubits, wherein several layers of 2-D cluster states are active at the same time, which will allow us to simultaneously perform QCN and distributed quantum computing. Moreover, when several 2-D cluster states are run in parallel on the same set of network nodes, we will be able to reconfigure the QCN as needed. This can be done with the help of the SDN concept. The SDN has been introduced to separate the control plane and data plane, manage network services through the abstraction of higher-level functionality, and implement new applications and algorithms efficiently. It has already been studied to enable the coexistence of classical and quantum communication channels. Our SDN-based QCN architecture is composed of three layers, namely an application layer, a control layer, and a QCN layer. Users send their requests from the application layer with the help of the northbound interface to the SDN controller. The SDN controller allocates the QCN resources with the help of its global map through the southbound interface. The QCN layer would be composed of dense wavelength-division multiplexing (DWDM) FSO/single-mode fiber (SMF)/few-mode fiber (FMF) links and QCN nodes. Any two nodes in the QCN can communicate through either through a dedicated SMF/FSO/FMF link or through a wavelength channel. The SDN control should also determine sequence of measurements to be performed in order to establish desired EPR pairs. To deal with time-varying channel conditions over heterogeneous links, we should adapt the system configuration based on both application requirement and link condition. 

## 3. Extending the Distance between Nodes in QCN

**Figure 4:**
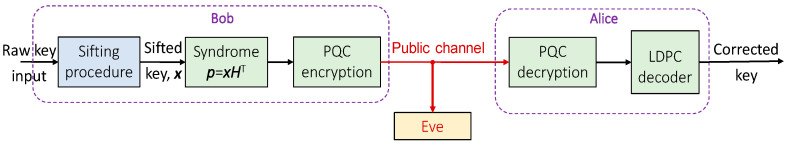
Illustration of post-quantum cryptography-based information reconciliation.

The DV-QKD can be used to build QKD networks, as discussed in the introduction. Unfortunately, the DV-QKD is affected by the deadtime of SPDs. Moreover, even if Eve cannot get the key because DV-QKD is used, she can prevent parties from creating secure keys, which is similar to the Denial of Service (DoS) attack. Further, since SKRs for DV-QKD are low, the quantum key pool, storing the secure keys, will often be empty, hampering the operation of QKD networks. To solve for this problem we propose to use the hybrid QKD-PQC protocols, in which QKD is used for raw key transmission and PQC in information reconciliation to reduce the leakage during the error reconciliation stage, which is illustrated in Figure 4. As mentioned in the introduction, the PQC is typically referred to in various cryptographic algorithms that are thought to be secure against any quantum computer-based attack. Unfortunately, the PQC is also based on unproven assumptions and some of the QPC algorithms might be broken in the future by developing advanced quantum algorithms. For this reason we propose to use the PQC algorithms only in the information reconciliation phase so as to limit the leakage due to transmission of parity bits over an authenticated classical channel (in conventional QKD). The quantum algorithms to be developed (not yet known), which will be capable of breaking the PQC algorithms, will have certain complexity expressed in terms of the number of operations *L*. By ensuring that the number of parity bits *N–K* is shorter than the number of secure PQC bits log_2_*L*, the proposed cryptographic scheme will be secure. Evidently, the proposed cryptographic scheme exploits the complexity of corresponding quantum algorithms used to break the PQC protocols. Given that the McEliece cryptosystem based on quasi cyclic (QC)-low-density parity-check (LDPC) coding is straightforward to implement as shown in [24], whereas the corresponding LDPC encoders and decoders have been already implemented in field-programmable gate array (FPGA) [25], it represents an excellent candidate to be used for the transmission of parity bits in the TF-QKD scheme. As an illustration, the secret fraction that can be achieved with the BB84 protocol is lower bounded by [1]:

(1) $$r=q_{}^{\left(Z\right)}\left[1-h_{2}\left(e_{}^{\left(X\right)}\right)\right]-q_{}^{\left(Z\right)}f_{e}h_{2}\left(e_{}^{\left(Z\right)}\right),$$

where $q_{}^{\left(Z\right)}$ denotes the probability of declaring a successful result when Alice sent a single-photon and Bob detected it in the *Z*-basis, *f_e_* denotes the error correction inefficiency (*f_e_* ≥ 1), *e*^(X)^ [*e*^(Z)^] denotes the QBER in the X-basis (*Z*-basis), and *h*_2_(*x*) is the binary entropy function $h_{2}\left(x\right)=-x\log_{2}\left(x\right)-\left(1-x\right)\log_{2}\left(1-x\right)$. The second term *q*^(Z)^*h*_2_[*e*^(X)^] denotes the amount of information Eve was able to learn during the raw key transmission, and this information can be removed from the final key during the privacy amplification phase. The third term *q*^(Z)^*f_e_ h*_2_[*e*^(Z)^] represents the amount of information revealed during the error correction stage. By sending the parity bits over the PQC channel this term can be effectively eliminated and the SKR can be increased.

**Figure 5:**
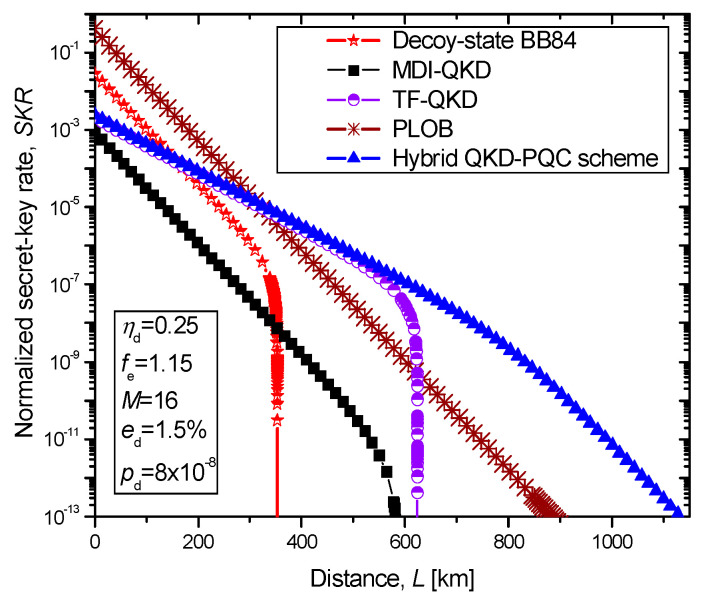
Proposed hybrid QKD-PQC scheme against MDI-QKD and TF-QKD in terms of secret-key rate vs. distance, assuming that ultra-low loss fiber is used.

By using this approach, as illustrated in Figure 5, the transmission distance between two nodes in QCN can be significantly extended. Here we provide comparisons of the joint TF-QKD-McEliece encryption scheme against the phase-matching (PM) TF-QKD protocol introduced in [26], the MDI-QKD protocol [6], and the decoy-state-based BB84 protocol [27]. The system parameters are selected as follows: the detector efficiency *η*_d_ = 0.25, reconciliation inefficiency *f*_e_ = 1.15, the dark count rate *p_d_* = 8 × 10^−8^, the misalignment error *e*_d_ = 1.5%, and the number of phase slices for PM TF-QKD is set to *M* = 16. Regarding the transmission medium, it is assumed that recently reported ultra-low-loss fiber of attenuation 0.1419 dB/km (at 1560 nm) is employed [28]. In the same Figure, the Pirandola–Laurenza–Ottaviani–Banchi (PLOB) bound on a linear key rate is provided as well. Both PM TF-QKD and joint TF-QKD-McEliece encryption schemes outperform the decoy-state BB84 protocol for distances larger than 162 km, while simultaneously outperforming the MDI-QKD protocol for all distances, and exceed the PLOB bound at a distance of 322 km. The PM TF-QKD protocol can achieve the maximum distance of 623 km. The proposed joint TF-QKD-McEliece encryption scheme is able to achieve the distance of even 1127 km, thus significantly outperforming all other schemes. Even though the operating wavelength was 1560 nm, other suitable wavelengths such as 2 μm and 3.9 μm can be used as well.

Now, by connecting the *base stations* to the nodes in the proposed QCNs, we can provide the unconditional security to the 5G+/6G wireless networks. By organizing the base stations in a quantum optical mesh network and employing the proposed hybrid QKD-PQC concept we can provide unconditional security to a large number of users. The Internet of Things (IoT) architecture will comprise widely distributed nodes connected via different types of channels to enable new functionalities in communication, sensing, and computing. Communication security in such a giant network is of paramount importance. Our proposed QCNs will underpin the unconditional physical-layer security of the IoT given that it will allow any two arbitrary nodes to securely transmit data at a high rate via an optical link. Critically, the security of such a network will not rest upon the trusted-node assumption, and a compromised node will not affect the security of other nodes. As such, the proposed QCNs will lead to a substantially stronger security level for the IoT. To enable security for future 6G wireless networks at a reasonable cost, the proposed joint satellite–terrestrial QCN can be based on the Cubesat satellites. 

For satellite-to-satellite quantum communications, in addition to the proposed hybrid QKD-PQC concept, it also possible to employ our recent restricted eavesdropping concept [29], which offers a significant increase in SKRs. This concept was presented in the ICTON 2020 paper [30]. Alternatively, the hybrid QKD can also be applied [13].

## 4. QCN under Development

**Figure 6:**
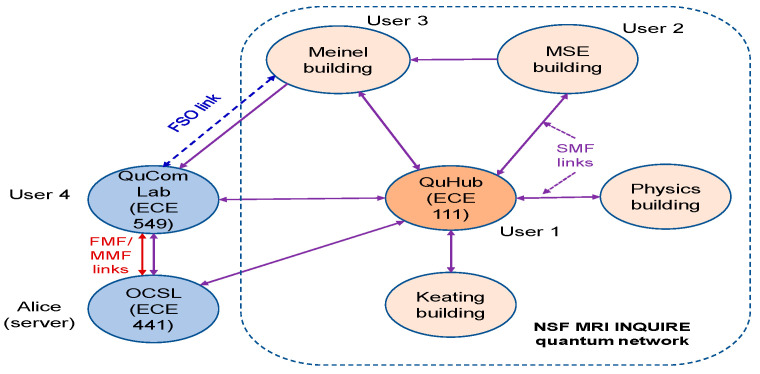
Terrestrial quantum communication network to be developed at the University of Arizona.

**Figure 7:**
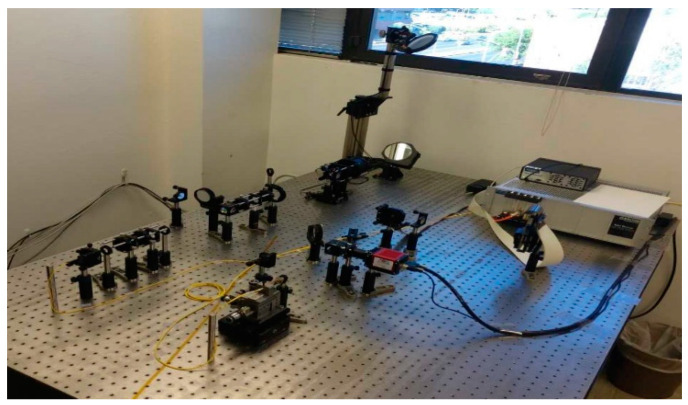
Free-space optical transceiver used in outdoor FSO link.

The terrestrial QCN to be developed at the University of Arizona is shown in Figure 6; it will exploit the existing NSF MRI INQUIRE quantum network, representing the quantum hub (QuHub) to share entangled photons and SPDs among different labs across the campus. The outdoor FSO bidirectional link, connecting the Electrical and Computer Engineering and Optical Sciences buildings, has already been established, with the FSO transceiver shown in Figure 7. We will also create the mesh network as well as the hybrid network composed of mesh, optical star, and ring network segments. The deployed heterogeneous QCNs will allow us to test novel quantum-networking theories and develop experimental tools for counteracting various channel impairments. To deal with atmospheric turbulence effects, the adaptive optics (AO) subsystem, composed of a wavefront sensor (WFS) and deformable mirror will be used. The AO will be combined with adaptive LDPC coding. 

To provide global coverage, we envision a scenario in which disconnected terrestrial QCNs, such as the one shown in Figure 6, are coupled through the LEO satellite quantum network. We have recently shown that a Bessel–Gaussian (BG) beam, carrying an orbital angular momentum mode, exhibits better tolerance to atmospheric turbulence effects compared to Gaussian beams for distances up to a few kilometers [31]. However, for LEO satellite-to-ground QuCom links, BG beams diffract much faster than Gaussian beams for such long-distance applications. Hence, we need to use pure Bessel beams to overcome this problem, as we have shown in our recent paper [32]. To enable robustness against turbulence encountered by FSO links, the envisioned quantum satellite QCN should communicate to ground nodes only through the LEO satellite-to-ground links, exhibiting a vertical downlink profile through vacuum followed by a turbulence layer with altitude-dependent strength. In principle. MEO/GEO satellite QCNs can be created above LEO QCNs to provide the planetary coverage.

## 5. Concluding Remarks

To enable the next generation of quantum-enabled cyber security systems, we proposed a quantum network of satellites that will provide the global coverage. The quantum satellite network will be composed of quantum subnetworks comprised of LEO satellites. Some of these LEO satellite-based quantum subnetworks will be connected to a subnetwork of MEO satellites. The MEO satellite subnetworks will then be interconnected to the global network of GEO satellites. The LEO/MEO satellites will also be used to interconnect terrestrial quantum networks. Each quantum communication subnetwork will be based on the cluster state concept. This quantum global network will allow us to establish EPR pairs between any two nodes in the global network. It can also be used to distribute the entangled states for quantum-sensing applications and to enable distributed quantum computing on a global scale.
